# The Relationship between Health Disparities, Psychosocial Functioning and Health Outcomes in Pediatric Hematology-Oncology and Stem Cell Transplant Populations: Recommendations for Clinical Care

**DOI:** 10.3390/ijerph17072218

**Published:** 2020-03-26

**Authors:** Evrosina I. Isaac, Andrea R. Meisman, Kirstin Drucker, Stephanie Violante, Kathryn L. Behrhorst, Alfonso Floyd, Jennifer M. Rohan

**Affiliations:** 1 Virginia Commonwealth University School of Medicine, 1201 E. Marshall St #4-100, Richmond, VA 23298, USA; kirstin.drucker@vcuhealth.org (K.D.); jennifer.rohan@vcuhealth.org (J.M.R.); 2 Children’s Hospital of Richmond at Virginia Commonwealth University, Richmond, VA 23219, USA; violantes@mymail.vcu.edu (S.V.); behrhorstkl@vcu.edu (K.L.B.); floydal3@vcu.edu (A.F.); 3 Division of Adolescent and Transition Medicine, Cincinnati Children’s Hospital Medical Center, Cincinnati, OH 45229, USA; andrea.meisman@cchmc.org; 4 Department of Psychology, Virginia Commonwealth University, Richmond, VA 23284, USA; 5 Massey Cancer Center Virginia Commonwealth University, Richmond, VA 23298, USA

**Keywords:** health disparities, cancer, sickle cell disease, bone marrow transplant

## Abstract

Not only do racial and ethnic minority children and adolescents with chronic illness experience disparities in health status and health outcomes, they also experience significant healthcare disparities, including differences in healthcare coverage, access to care, and quality of care. It is well known that the interaction between psychosocial functioning, health behaviors and ethnic and racial disparities, ultimately leads to worse health and psychosocial outcomes in pediatric and AYA chronic illness patient populations, including increased rates of morbidity and mortality. Investigating the impact of racial and ethnic factors on health outcomes, and strategies for reducing these disparities, is of the utmost importance, specifically in life-threatening conditions like cancer and sickle cell disease. This commentary underscores the relative importance of identifying factors that could reduce disparities between minority and non-minority populations. This present paper will focus on the dynamic relationships between health disparities, psychosocial factors and health outcomes within pediatric cancer, sickle cell disease and bone marrow transplant populations, and will offer recommendations for healthcare professionals working with these vulnerable patient populations. The primary goal of this commentary is to provide recommendations for enhancing cultural competency and humility for those working with highly vulnerable patient populations.

## 1. Introduction

According to the U.S. National Center for Health Statistics, a chronic disease or illness is defined as a disease that lasts at least 3 months, with an expected duration of at least one year [1,2]. It is estimated that 1 in 5 children in the United States will suffer from a chronic illness with prevalence rates increasing each year [1,2].

Chronic illnesses can be biological, psychological, or cognitive in nature, including, but not limited to, depression, anxiety, autism, attention deficit hyperactivity disorder, autoimmune disorders, asthma, arthritis, HIV, cancer, sickle cell disease and other blood disorders [1,2]. It is notable that chronic illnesses disproportionately affect racial and ethnic minority children and adolescents, who are 1.5 to 2 times more likely to be diagnosed with a chronic illness than their white counterparts [3]. An ethnic and racial minority group is defined as a group of people who are disadvantaged as a result of the group they belong to or identify with, based on the color of their skin, their ethnic origin, or their geographical region [4]. Not only do racial and ethnic minority children and adolescents with chronic illness experience disparities in health status and outcomes, such as differences in disease burden, injury, or mortality [4,5], but they also experience significant healthcare disparities, including differences in healthcare coverage, access to care and quality of care, which subsequently impacts upon their health outcomes [4,5,6]. Investigating the impact of racial and ethnic factors on outcomes, and strategies for reducing these disparities, is of the utmost importance, specifically in life-threating conditions like cancer, sickle cell disease and other blood disorders.

Health disparities are defined as an avoidable health outcome that is attributed to inequalities due to unequal access to resources (e.g., differences in disease burden, injury, or mortality between groups) [5]. On the other hand, healthcare disparities are defined as differences in health care coverage, access to care, and quality of care between different ethnic/racial groups [6,7,8]. Several organizations have proposed initiatives that investigate the multifaceted inequalities observed in healthcare, with a common goal of identifying those disparities that are both unfair and avoidable [9]. In addition, some have emphasized the importance of identifying these disparities in order to screen individuals more susceptible to a disease sooner [10]. In fact, Congress requested that the Institute of Medicine assess differences in health care quality received by racial/ethnic minority patients compared to non-minority patients [6,11]. The key findings from this report indicated that racial and ethnic disparities in healthcare exist and occur in the context of broader historic and economic contexts [6]. These findings were summarized along with possible sources of disparities, and recommendations on how to eliminate these unnecessary inequalities in health outcomes between different ethnic/racial groups [11]. Furthermore, more recently, race has been defined by the American Academy of Pediatrics as a core determinant of child health outcomes in a recent policy statement on the impact of race on both child and adolescent health [12].

The United States Department of Health and Human Services Office of Minority Health has defined culture as “integrated patterns of human behavior that include the language, thoughts, communications, actions, customs, beliefs, values, and institutions of racial, ethnic, religious, or social groups” [4]. This definition emphasizes that culture should be defined distinctly from race/ethnicity, and not used interchangeably. Often, just knowing a patient’s ethnic, racial, or cultural background does not predict their beliefs and attitudes towards their healthcare. Moreover, it would be important to distinguish which cultural traits are being studied, and what measures would be appropriate [4]. As a result, the studies that conclude that health outcomes or behaviors solely differ by race, ethnicity, or acculturation, have failed to account for unique cultural traits within racial and ethnic groups [4]. Unfortunately, the measurement of cultural traits exclusively does not always assess the differences in health behaviors and outcomes that are often accounted for when considering cultural, ethnic and racial factors together.

In summary, the interaction between contextual factors, including psychosocial functioning, health behaviors, and ethnic and racial disparities, are shown to be associated with worse health outcomes and psychosocial outcomes, and increased rates of morbidity and mortality in pediatric and AYA chronic illness patient populations [3,4,5,6,7,8,10,11,12,13,14,15,16,17,18,19,20,21,22,23,24,25,26]. It is well demonstrated that individual- and family-level factors, including ethnicity/race, environmental factors, cultural factors, psychosocial stressors, resilience and family dynamics, are all potential predictors of disparities in health status and ultimately worse health outcomes in pediatric chronic illness. This commentary underscores the relative importance of identifying these potentially modifiable and non-modifiable factors that could reduce disparities between minority and non-minority patient populations, especially in pediatric populations with life-threatening diseases, such as cancer, sickle cell disease and related blood disorders.

The present paper will focus on the dynamic relationships between health disparities, psychosocial factors, and health outcomes within these pediatric illness populations, and will offer recommendations for healthcare professionals working with these vulnerable patient populations. Given the multifaceted complexities of managing health-related and culturally-related factors that may influence health disparities among pediatric patients with a chronic illness, this paper provides careful considerations of the importance of cultural competence (i.e., the ability to work with people of different ethnic/racial backgrounds, cultural backgrounds and groups), and cultural humility (i.e., the ability to honor others’ backgrounds and beliefs) in working with ethnic/racial minority pediatric patients and families. Thus, the primary goal of the present paper is to provide recommendations for enhancing cultural competency and humility for those working with highly vulnerable ethnic/racial minority pediatric patients and families.

## 2. Management of Chronic Illness Can be a Multifaceted Challenge for Pediatric Patients

The impact of chronic illness on patients and families is multifaceted. Regardless of race or ethnicity, all individuals with a chronic illness may experience significant impacts in their functional activity, cognitive functioning, emotional functioning and social growth and development [2]. Additionally, patients with a chronic illness may suffer from physical deficits, such as decreased ambulation or ambulatory difficulties, and fine or gross motor deficits, which have the potential to ultimately impact their overall quality of life and psychological well-being [2]. Given the impact that chronic illness has on children and adolescents, individuals with chronic illness may report a significant change in their social roles compared to healthy peers, and/or may report that deteriorations or changes in their medical status results in significant lifestyle changes, altering how they function at the individual- and family-level.

Furthermore, having a chronic illness may require patients to take medications or make dietary changes, use medical equipment or assistive devices like wheelchairs or walkers, and/or require the need for personal assistance, including increased support from parents and other caregivers. Patients may also need additional academic supports, including educational resources like IEPs and 504 plans, and/or vocational services, such as job coaches, specialized vocational training, etc. The need for additional resources and supports can greatly impact psychological well-being and quality of life. Furthermore, while adhering to treatment regimens and engaging in positive self-management behaviors is important for optimal health outcomes, the increased burden and time required to care for a chronic illness, may ultimately impact psychological functioning, general well-being and quality of life for both the patient and the entire family system.

### 2.1. Social Determinants of Health and the Relationship to Psychosocial and Health Outcomes

Many programs have been established to address various social determinants of health, and the inequalities that they create within the medical realm. An example of these programs include clinical guidelines, screeners and other standardized policies focused on identifying factors associated with disparities. The American Academy of Pediatrics has put forth Bright Futures, a national health promotion program. This program has offered guidelines to guide healthcare providers through clinic visits. Uniquely, these guidelines highlight the importance of looking at children and their health in the context of their families and communities [27]. There are also screeners in place in primary care clinics to specifically look at psychosocial issues. Increasingly, screeners have been developed to look at basic needs, such as food and employment, at routine pediatric medical visits. These have increased appropriate physician referrals and connections between patients and families with community resources [27]. In the primary care setting, intervention is becoming more vital as a result. Two examples of programs that emphasize primary care intervention are Reach Out and Read, and Health Leads. The Reach Out and Read program uses the relationship that pediatricians have with families, and utilizes the physician providers as counselors to help combat patients’ learning problems [27]. Similarly, Health Leads creates an avenue for families to connect with community resources by placing undergraduate volunteers in the waiting rooms of urban clinics [27].

It has been known that often socioeconomic conditions are at the forefront of health inequalities. As a result, social policies have been brought to attention that may impact health. Policies regarding wages and income can help people in low income brackets attain increased financial security. The Earned Income Tax Credit is one example of a program that has helped in this realm [17]. In addition, home visit programs such as the Nurse–Family Partnership allow for increased access to health needs, as well as an assessment of family needs and coordinating various services [17]. To further decrease health inequalities by tackling socioeconomic conditions, future policies need to strengthen assessment and action on health-improving social policies, expand policies to facilitate health behaviors, and improve access and the financing of health care services [17].

### 2.2. Individual, Familial, Environmental, and Cultural Factors May also Influence Psychological Functioning and Health Outcomes of Patients with a Chronic Illness

The patient’s environment as one determinant of health outcomes is well-researched in the context of the stress-exposure disease framework. This framework suggests that residential segregation is a potential cause of racial and ethnic health disparities [19]; such that, racial and ethnic groups often disproportionately live in high-poverty areas, which are more likely to be exposed to health risk factors, such as pollution, and individuals in these areas are less likely to have access to much needed health resources, including healthcare [3,4,19]. These increased disparities will increase the community-level vulnerability of these patient populations. The stress-exposure disease framework suggests that this higher community-level vulnerability then increases those individual psychosocial and biological stressors, ultimately resulting in a higher prevalence of chronic illnesses in the setting of limited access and resources [19]. On the other hand, healthcare resources are readily available to non-minority patient populations who are not living within the poverty-level [19]. It is notable that community-level and individual-level stressors disproportionately experienced by racial and ethnic minorities often interact, thus increasing disparities in these vulnerable patient populations.

There are several other factors that may explain some of the observed differences between minority and non-minority patient populations, leading to disparities in health outcomes, which include [4,6,13,14,15,16,17,18,19,20,21,22,23,24,25,26]: psychosocial functioning (individual, family); differences in treatment adherence including adherence to prescribed medications and frequency of medical follow-up; socioeconomic status; decreased access to much needed resources, such as transportation and prescription benefits; cultural differences; and decreased access to healthcare, especially for those who live in poverty-stricken environments or rural areas. For example, Pritchard et al. [21] found that non-minority children have better medication adherence and clinic visit attendance compared to minority children. Cultural or socio-cultural differences refer to differences in beliefs, attitude, language, thoughts, communications, actions, values, religious beliefs, racial differences, ethnic differences, social groups and/or customs [4]. In the setting of health behaviors, cultural differences may be defined as common or shared values, beliefs, or practices that are directly related to a specific health behavior [4]. Impacts of cultural differences may include an unfamiliarity with the medical system due to geographic distance from healthcare practices, misunderstandings about the purpose of treatment or cause of illness, erroneous (or not so erroneous) beliefs about medication and treatment (e.g., “medication is harmful,” “I/we trust in God to cure disease.”), and a general distrust in medical providers and/or the medical system, which can ultimately lead to worse outcomes, increasing the potential for additional disparities between minority versus non-minority populations [4,18,27]. Furthermore, cultural differences in caregiver–child interaction styles may contribute to increased family conflict, and hence impact health behaviors like treatment adherence, ultimately impacting short- and long-term health outcomes [21,28]. For example, parents who are high in negative affect may be harder on children and adolescents with chronic illness who do not take responsibility for their own illness, particularly when children and adolescents do not adhere to parental commands, such as reminders to take medications, thus leading to increased nonadherence behaviors [29].

### 2.3. Systemic Factors Leading to Healthcare Disparities

It is important to note that there are also upstream systemic factors that cause healthcare disparities. For example providers, regardless of specialty and level of training, have been shown to have implicit and sometimes explicit biases towards those of Hispanic and African American backgrounds [30]. These biases could potentially lead to a different quality of care being offered to patients, and thus indirectly or directly causing healthcare disparities. A potential problem faced by changing the providers’ perspective is that many may not realize that they hold these biases. In a study with surgeons from around the country, only one third of the surgeons recognized that healthcare disparities even existed in surgical care [31]. In addition, hospital system factors can impact healthcare received by groups. For example, the distance to the closest hospital may prevent patients of low socioeconomic status to seek out care, due to transportation issues or having to work [32]. Patients who are of non-English-speaking backgrounds, may not be able to seek care if these institutions do not employ on-site translators [32]. As a result, large-scale system factors may also drastically impact disparities experienced by patients.

In summary, managing a chronic illness is both complex and multi-faceted. Regardless of the disease type, disease status, or prescribed treatment, all pediatric patients and their families are presented with a unique set of challenges that places additional demands on the individual and family system. We will now describe the challenges encountered across pediatric cancer, sickle cell, stem cell/bone marrow transplant and pediatric cancer survivors, and offer recommendations for improving health behaviors and ultimately health outcomes in these vulnerable patient populations. We have focused this commentary on the aforementioned patient populations due to the limited and often contradictory findings of the pediatric literature investigating health disparities on parent–child dynamics, psychosocial factors, and health outcomes within these specific groups. In addition, the complicated treatment and post-treatment course of these patients makes them more susceptible to being impacted by social disadvantages, which will ultimately impact health outcomes. This commentary provides an overview of disparities often observed within these patient populations, and recommendations for clinicians to help reduce disparities, which will improve outcomes.

## 3. Health Disparities and Pediatric Oncology Patient Populations

The most common cancers diagnosed in children and adolescents from infancy to age 19 years are leukemias, brain and central nervous system (CNS) tumors and lymphomas [33]. Survival rates for pediatric cancer have exponentially increased over the past 20 years [33]. This is largely due to the use of intensive chemotherapy regimens, mostly given parenterally. Despite the increased availability of molecular agents known to have positive outcomes, including increased survival rates, there continues to be a higher than expected mortality rate in pediatric cancer. Previous research in pediatric cancer identified several individual- and family-level factors and social factors, including, but not limited to, patient gender, age, ethnicity/race, disease knowledge, health beliefs, coping and parent–child relationship dynamics that are known to directly or indirectly impact health behaviors, and ultimately health outcomes [20,21,22,34,35,36,37,38].

Significant ethnic and racial disparities in clinical outcomes for pediatric patients diagnosed with cancer are well documented, including, but not limited to [4,13,14,17,20,21,22,23,24,34,39]: cultural or socio-cultural differences, socioeconomic status, access to care, knowledge about cancer diagnosis and treatment, geospatial location, and disease biology (such that minority children may have an increased risk for medication toxicities compared to non-minority peers). In a large, longitudinal study of 575 children with leukemia, researchers found that survival rates in high poverty areas were significantly lower (85%) than those patients who were living in low poverty areas (92%) [40]. It is likely that those individuals who live in high poverty areas are more likely to have limited access to medical services, and have lower health literacy, which results in limited knowledge about the need to seek medical care, and thus a worse prognosis over time [40,41].

Furthermore, even when seeking medical care and receiving a diagnosis of cancer, under-resourced patients are unlikely to fully understand the diagnosis and treatment protocols compared to high-resourced families, which ultimately leads to increased risk of poor self-management behaviors, poor adherence to treatment, and worse health outcomes. Bhatia et al. [22] examined rates of 6-mercaptopurine (6MP) nonadherence, which is a daily oral chemotherapy medication for cancer, in Hispanic versus non-Hispanic patients. They found that being of Hispanic ethnicity, being older than age 12 years old, and/or being in a single parent household, resulted in significantly lower rates of 6MP medication adherence, and hence worse outcomes. These researchers also found that a medication adherence ≤95% was associated with a higher risk of disease relapse [20].

Under-resourced families also have a higher risk for psychosocial impairments (e.g., anxiety, depression, financial stressors, limited social supports), which has been shown to negatively impact health behaviors and health outcomes over time [13,14,17,20,28,38,41,42]. Psychosocial stressors often put competing demands on patients’ and parents’ lives that pose inevitable barriers to treatment success, including less than optimal self-management and health behavior patterns from new diagnosis to survivorship [13,14,17,20,28,38,41,42]. Moreover, health beliefs, neurocognitive functioning, psychosocial functioning, associated treatment burden, and problems in communication and problem-solving strategies, pose salient barriers to developing and refining more adaptive health behaviors [21,28]. Prior research also found that patients whose caregivers completed less education and/or identified increased financial difficulties had significantly greater risk for psychosocial problems [41].

Parents, regardless of their racial or ethnic identity, also reported wanting as much detailed information as possible about their child’s disease, treatment and prognosis [17,20,43]. However, disparities were observed in how physicians delivered information to minority patients and families, with the assumption that African American and Hispanic patients, parents and families were less interested in receiving detailed information compared to Caucasian and non-minority peers [43]. An inaccurate perception of what patients or families want to hear solely based on their presentation, including their racial/ethnic identity or level of health literacy, can create a great obstacle in developing a trusting partnership between parents and healthcare providers who are caring for their child. It was suggested that physicians should regularly assess what patients and parents understand, and determine how much information they would like to have shared with them to prevent miscommunication or unspoken biases [43]. This should be routinely re-addressed and assessed throughout the care of the child to enhance trust and decision-making.

While managing oncology disorders presents a set of unique challenges for patients and families, recognizing potential cultural barriers that could reduce disparities is paramount for reducing morbidity and mortality rates in pediatric cancer. We will now describe potential challenges that patients and their families may encounter following cancer treatment.

## 4. Health Disparities and Pediatric Cancer Survivors

Patients who achieve disease remission and complete treatment for pediatric cancer will successfully move on to the next phase of cancer treatment, i.e., long-term follow-up or survivorship. As a cancer survivor, continued medical surveillance is essential to monitor for early signs of disease relapse, recurrence, and late-term effects like cognitive dysfunctions, obesity and cardiovascular issues [44,45,46,47]. Disparities observed during cancer treatment also are frequently observed during the survivorship phase. For example, patients with financial difficulties, or those coming from a low socioeconomic status, may have increased nonadherence to surveillance recommendations, due to lack of healthcare insurance, access to healthcare, or limited resources, which poses an increased risk for developing adverse outcomes. In a previous study, lower socioeconomic status predicted decreased knowledge of survivorship recommendations, and hence worse health behaviors over time, which could lead to worse health outcomes and an increased risk of adverse effects long after treatment ends [44,45,46,47].

It has become increasingly important to also monitor psychosocial late effects post-treatment, in addition to medical late effects. Although the findings are mixed, there has been some evidence to show that childhood cancer survivors are at an increased risk for developing depression, anxiety and post-traumatic stress, compared to peers who were not diagnosed with cancer [44,45,48]. Less is known about how the receipt of psychological services during cancer treatment prevents or mitigates the risk of developing psychological symptoms post-treatment. Furthermore, patients’ anxiety post-treatment can be exacerbated by attending surveillance visits due to increased fears and worries about disease recurrence or other medical complications. It is believed that this added anxiety may contribute to nonadherence to clinic visits [47]. Thus, it is important for providers to be able to recognize how disparities can impact future care to reduce the impact of disparities on these vulnerable pediatric patient populations long after treatment ends. We will now describe potential challenges that sickle cell patients and their families may encounter when managing a chronic illness and interacting with healthcare providers and the larger healthcare system, which demonstrate both unique challenges within this population, but also similarities with the oncology population.

## 5. Health Disparities and Sickle Cell Disease Patient Populations

Sickle cell disease is an autosomal recessive disorder, which affects 100,000 Americans, including one of every 365 African American births, and one of every 16,300 Hispanic-American births [49]. The sickle cell trait is a less severe form of this disease, in which one inherited gene codes for normal hemoglobin, and the second inherited gene codes for sickle hemoglobin. In the United States, one in 13 African American births are affected by this sickle cell trait [49]. Sickle cell disease affects predominantly minority patients, and it has been suggested that interventions should align with the cultural needs of this patient population [49,50,51,52,53,54]. It should be emphasized that self-esteem, racial pride and racial discrimination are all potential factors that impact the health behaviors and health outcomes of patients with sickle cell disease [55]. Therefore, cultural competency and cultural humility play an essential part for healthcare providers working with this patient population, including providers who are developing innovative therapeutic interventions for improving psychosocial and health outcomes for this patient population.

Research has shown that the implicit or explicit biases of healthcare providers impacts the quality that care patients with sickle cell disease receive [56]. Not only could the quality of care be impacted, but a general mistrust in the medical system may be created as a result of these biases, which ultimately leads to skepticism of potential medical interventions that are recommended by healthcare providers. This distrust and skepticism may ultimately result in suboptimal health outcomes [52,53,56]. Currently, hydroxyurea is one of the few approved medications for pediatric patients with sickle cell that can help with reducing the frequency and intensity of sickle cell pain crises. Despite the benefits of this medication, nonadherence to this medication is high [50,51,57,58,59]. While medication nonadherence often is multi-faceted, with common barriers to adherence identified across all pediatric chronic illness populations (e.g., forgetting, illness severity, symptom severity, treatment burden, knowledge, disease duration, side effects, developmental factors, psychological factors, peer influences and dislike/distrust of “authority”) [38], there may be additional unique challenges with this particular medication when used with this specific patient population. Nonadherence to hydroxyurea may stem from beliefs about the medication’s safety, and a general distrust in the medical team (e.g., “this is a chemotherapy medication, why is my child taking medication for cancer”) [50,51,57,58,59]. Even with strong supportive research on the safety of hydroxyurea, some patients and families are hesitant to start the treatment until a severe sickle crisis occurs [50,51,57,58,59]. This is problematic, because this medication is most effective when used in a preventative manner, meaning that the medication is taken every day, regardless of the frequency or intensity of sickle cell pain. Using it on an “as needed” basis eliminates the benefits of the preventative treatment, reduces the potential effectiveness of the medication, and may ultimately lead to discontinuation because of the minimum benefit observed by the patient and family [60].

There have been correlations found between children’s coping styles, healthcare utilization, and the report of psychological distress in pediatric sickle cell patient populations [52,56,61]. For example, patients with sickle cell disease, whose coping style is characterized as being negative and passive, will have an increased utilization of health care resources, and increased psychological problems in comparison to patients who have positive and active coping styles [49,62]. In fact, Klitzman et al. [49] showed that participation in regular, daily family routines and more open family communication were protective factors for managing sickle cell disease, ultimately reducing disease morbidity. In contrast, there were differences between patient-reported versus parent-reported medication adherence. Thus, psychological interventions promoting health behaviors in this population should incorporate adherence promotion [49].

In a cohort of African American adolescents with sickle cell disease, Schwartz et al. [61] identified ten components to consider when developing and providing effective interventions with this patient population, which included a focus on family-based interventions, emphasis on empowerment, recognition of stress related to ethnic minority status, identification of stress related to socioeconomic status, inclusion of culturally sensitive content, awareness of stigma attached to mental health problems, provision of community or home-based interventions, flexibility in scheduling, and ongoing training in cultural sensitivity with the clinical and research teams due to possible mistrust of the healthcare system, medical providers and research participation [61]. Clinicians should also recognize the importance of the extended family, and incorporate additional caregivers in interventions as relevant. Given the homogeneity of the sickle cell patient population, and the higher rates of the ethnic/racial diversity of healthcare providers working with this patient population, cultural competence and humility of all healthcare providers, regardless of their own ethnic/racial identity is of the utmost importance.

## 6. Health Disparities and Bone Marrow Transplant Patient Populations

Bone marrow transplants can be used for a variety of chronic illnesses, including but not limited to, sickle cell disease, acute myeloid leukemia (AML), chronic myeloid leukemia (CML), relapsed acute lymphoblastic leukemia (ALL), and other relapsed cancers and lymphomas. In some cases, bone marrow transplants are a standard of care, such as for AML; however, bone marrow transplants may also be used as the last attempt to cure a patient of their disease. Despite the potentially lifesaving and curative nature of a bone marrow transplant, treatment adherence is still a problem with this especially vulnerable patient population, leading to increased rates of morbidity and mortality [63,64]. Thus, identifying factors contributing to disparities is imperative when working with this patient population.

While the ethnic and racial disparities that are observed in this patient population are synonymous with the other populations described earlier, bone marrow transplants for ethnically- and racially-diverse patient populations also have unique complexities. Be The Match estimates that the likelihood of an African American patient finding an unrelated donor match is ~23%, Asian or Pacific Islander is ~41%, Hispanic or Latino is ~46% and American Indian or Alaska Native is ~57% [65,66]. One of the criteria for matching bone marrow transplant recipients with potential donors is the human leukocyte antigen type (HLA). HLA markers, however, are also inherited, meaning only patients of the same ethic/racial background, or those who are related donors are more likely to be a successful match. As a result, ethnic minorities who need bone marrow transplants are automatically at a disadvantage [65,66]. Not only are minority patients at a disadvantage when identifying a donor, but patients are at increased risk for developing disease-related morbidity and mortality, including a higher risk of developing severe graft-versus-host disease post-transplant [67]. Furthermore, socioeconomic status can impact outcomes for patients receiving a bone marrow transplant. There are high costs associated with the transplant process and post-transplant care, which could incidentally result in increased disparities before or after transplant, even when disparities may not have existed before [63,66,67].

## 7. Cultural Competence in Pediatric Chronic Illness: Recommendations for Clinical Practice

Acknowledging cultural differences and practicing humility are effective strategies to mindfully engage with pediatric patients with chronic illnesses of all ethnic and racial backgrounds. Clinical interventions should incorporate culturally competent, evidence-based strategies from a diverse framework of well-established treatment modalities, such as cognitive behavioral therapy (CBT), health promotion, adherence promotion, interpersonal therapy, acceptance and commitment therapy (ACT) and motivational interviewing (MI). Interventionists should tailor their intervention, including utilizing a personalized intervention approach for the individual and family unit whom they are seeing that considers individual- and family level factors, cultural and environmental factors, and other factors that are known to increase disparities.

Successful intervention models with ethnically diverse patients and families should be informed by a framework of social cognitive theory, which posits that behavior is determined by individual beliefs, motivation to change, and social environmental factors [68], each of which can be potential barriers and facilitators to treatment success, and may increase the potential for disparities. A patient- or family-centered intervention that focuses on a discussion of problems that indirectly and directly influences health outcomes, can result in positive health behavior change [68]. Barriers such as peer relationships, interpersonal difficulties, and problematic family routines, that are potentially modifiable through brief psychological interventions and successful problem-solving within the family unit, should be one potential target of intervention. In addition, parental behaviors, such as monitoring and modeling of constructive problem-solving and encouragement have been shown to be important in health promotion interventions with children and adolescents and young adults (AYAs) [21]. Given the importance of peer relationships on psychological outcomes and the quality of life, interventions should also target the development of enhanced social support. Finally, intervention models that implement a person-centered and/or family-centered approach, designed to enhance family and interpersonal relationships, by promoting communication and collaboration between caregivers and children/adolescents, is warranted for successful behavior change and optimal health behavior patterns [27,69].

Additionally, health promotion interventions in pediatric chronic illness, specifically within hematology and oncology and bone marrow transplant populations, should also utilize tenants of sociocultural deterrent models and family-systems eco-developmental perspectives [10,14,15,17,20,22,23,24,25,26,70,71,72] that consider the stressors experienced by families of children and AYAs with cancer, sickle cell disease and related conditions, including those known with greater rates of health disparities. This type of intervention model considers stressors, such as marginalization from mainstream culture, and acculturation issues that are experienced by families from different cultural backgrounds, especially African American and Hispanic families [71,72]. This family-centered, eco-developmental model should address several issues during the treatment process, such as the empowerment of families’ cultural beliefs about cancer or chronic illness treatment, and enhanced communication with medical providers and each other [10,15,25,70,71,72]. The Familial Unidas model, which has been applied effectively to manage adolescent risk behavior in minority adolescents [71,72], also has special relevance for working with at-risk youth with chronic illnesses, such as those with sickle cell disease, who have higher rates of risky behaviors.

One tenet of CBT and other interventions that can be successful with reducing barriers and maximizing the facilitators of success is the use of problem-solving techniques to identify barriers in an effort to reduce potential disparities from impacting health behaviors and health outcomes. This can be an effective treatment modality to reduce disparities, as the approach includes teaching and reinforcing problem-solving skills in individuals and families, with a focus on enhancing behavioral competence, such as developing skills for healthy lifestyle management, and reducing barriers impacting the promotion of optimal health behaviors [29]. Consistent with problem-solving theory, the primary “problems” to be solved during interventions should involve identifying any barriers to adaptive/optimal self-management and health behavior patterns, and identifying specific areas that need improvement with a focus on reducing disparities [29].

The principles of problem-solving interventions have been empirically tested with patients of diverse backgrounds, including those with cancer and sickle cell disease and their parents [29].

Finally, cultural humility may be directly related to psychosocial factors and health outcomes. Rapport with patients and families may be negatively impacted if a clinician does not successfully practice cultural humility during all patient interactions. Negative rapport between providers and patients will likely impact health behaviors, and ultimately health outcomes. Cultural humility also includes being respectful of other cultures, including negating the use of alternative medicines, complementary treatment, or spiritual treatment [4,5,43]. As providers, it is very important for us to engage patients and families in a shared decision-making process, including understanding their rationale for wanting to postpone treatment or explore alternative options. As an integrated team, clinicians, patients, and families should explore these alternative options together to determine what might be the “best” possible solution for their care, while also maximizing their quality of life. If a medical provider argues with a patient or family regarding their wishes, or dismisses their beliefs, or attempts to immediately discuss evidence-based treatment modalities and options without considering their input, cultural beliefs and/or spiritual beliefs, the provider will likely lose whatever rapport was already established with that family, pushing that patient and family away from them, increasing distrust in the medical team and healthcare system, and ultimately putting the patient at a high risk for adverse health outcomes.

## 8. Conclusions

In summary, our review demonstrates the dynamic relationships between health disparities, psychosocial factors, and health outcomes within pediatric cancer, sickle cell disease and bone marrow transplant populations. Our commentary underscores the relative importance of identifying factors that could reduce disparities between ethnic/racial minority and non-minority populations. We reviewed several recommendations and evidence-based strategies, and interventions that can be utilized to reduce the adverse effects of conscious and unconscious biases when working with diverse patient populations. Utilizing these culturally competent strategies, and recognizing the importance of cultural humility in your work with vulnerable patients and families, will ultimately improve the health outcomes of all patients who are receiving care for their chronic illness.
